# Predicting the volatility of Chinese stock indices based on realized recurrent conditional heteroskedasticity

**DOI:** 10.1371/journal.pone.0308967

**Published:** 2024-10-18

**Authors:** Gongtao Zhang, Huanyu Zhao, Rujie Fan

**Affiliations:** School of Finance, China Academy of Financial Research, Southwestern University of Finance and Economics, Chengdu, China; Shanghai Jiao Tong University, CHINA

## Abstract

The realized recurrent conditional heteroscedasticity (RealRECH) model improves volatility prediction by integrating long short-term memory (LSTM), a recurrent neural network unit, into the realized generalized autoregressive conditional heteroskedasticity (RealGARCH) model. However, at present, there is no literature on the ability of the RealRECH model to fit and predict volatility in the Chinese market. In this paper, a study is conducted to test the in-sample explainability and out-of-sample prediction ability of the RealRECH model for the SSE50, CSI300, CSI500 and CSI1000 indices in the Chinese market and to determine whether it performs better than the RealGARCH model. The results of the in-sample analysis show that the RealRECH model not only provides better in-sample interpretability for all four indices but also captures the complex dynamics of time series volatility that the RealGARCH model cannot capture, such as long-term dependence and nonlinearity. The results of out-of-sample volatility prediction show that the RealRECH model better predicts the volatility of the CSI500 and CSI1000 indices but yields worse predictions for the SSE50 and CSI300 indices. Thus, the RealRECH model can be used for CSI500 and CSI1000 prediction.

## 1. Introduction

Improving predictions of financial time series volatility is important for the calculation of value at risk (VaR), option pricing and portfolio management. The effect of predicting the volatility of financial time series depends on whether the model can capture the characteristics of volatility. The traditional volatility model originated from the generalized autoregressive conditional heteroskedasticity (GARCH) model of Engle [1] and Bollerslev [2]. The disadvantage of this model is that it uses low-frequency data, so its prediction effect is poor. Hansen et al. [3] introduced high-frequency data into the GARCH model instead of low-frequency data and proposed the realized generalized autoregressive conditional heteroskedasticity (RealGARCH) model.

With the emergence of deep learning models and improvements in computing abilities, many scholars have begun to use neural networks (NNs) to predict volatility. Liu [4] and Bucci [5] compared the predictive ability of feedforward neural networks (FNNs) and recurrent neural networks (RNNs) with that of traditional econometric models, and they found that the predictive ability of deep learning models was better than that of traditional econometric models. However, when predicting the volatility of financial time series, although deep learning models can achieve better predictive performance than GARCH models, deep learning models lack economic explainability.

These models are still widely used in Chinese market research. In recent years, three kinds of models have primarily been used in Chinese market research: traditional volatility models, such as the GARCH model and its variants; isolated recurrent neural network (RNN) models; and models that combine neural network models with GARCH. For example, Cui et al. [6] used a GARCH model based on the T distribution and generalized error distribution (GED) to study whether volatility clustering and leverage effects exist in the Chinese stock market. The results of 12 Chinese stock indices showed that these characteristics do exist. Liu et al. [7] studied the role of high-frequency data in the volatility prediction of Chinese stock markets using the GARCH model with different distribution assumptions and found that the greater the frequency of the return series used is, the better the volatility prediction of the GARCH model. Furthermore, a GARCH model using 5-minute high-frequency data outperformed not only all other GARCH models with lower data frequencies but also other models based on realized volatility, such as the heterogeneous autoregressive (HAR) and autoregressive fractionally integrated moving average (ARFIMA) models.

Liu and Shi [8] used the component GARCH (CGARCH) model based on the assumption of a tempered stable distribution to study the volatility of the Shanghai Stock Exchange indices and found that the fitting ability of this model is better than that of the CGARCH model based on the assumption of a normal distribution, T distribution or GED. Luo [9] used three SSE 50ETF index training sets with different time spans and compared the volatility prediction performance of three deep learning models, namely, simple recurrent network (SRN), long short-term memory (LSTM) and gated recurrent unit (GRU) models, with that of the traditional GARCH model based on test sets. The results showed that in the long term, the deep learning model achieved better volatility prediction performance, but the stability of the model was obviously worse than that of the GARCH model, and its performance was highly dependent on the selection of the training set. Zhao et al. [10] compared the in-sample volatility prediction ability and out-of-sample predictive ability of the GARCH model, a simple recurrent network GARCH (SRN-GARCH) model and a statistical recurrent unit GARCH (SRU-GARCH) model in a study of four stock indices in the Chinese market; they found that both the SRN-GARCH model and the SRU-GARCH model yield better volatility explainability and predictive ability than the GARCH model for the CSI 500 and CSI 1000 indices but not other indices.

In general, as mentioned above, existing studies on the prediction of Chinese market volatility mainly used three types of models: the GARCH model and its variants, isolated RNN models, and models that combine neural network models with GARCH. At present, there has been no research on the use of the realized recurrent conditional heteroscedasticity (RealRECH) model to fit and predict the volatility of the Chinese market. This model was proposed by Liu et al. [11], and its details are described in Section 2. The question remains of how does the RealRECH model perform in the Chinese market in terms of in-sample volatility explainability and out-of-sample predictive ability? This is a question worth studying.

In this paper, we use the RealGARCH [3] and RealRECH [11] models to study the volatility of four stock indices in the Chinese market, namely, the SSE 50, CSI 300, CSI 500 and CSI 1000. The in-sample empirical results show that first, the RealRECH model yields larger in-sample marginal likelihood values for all four indices, which means that the RealRECH model provides better volatility explainability than does the RealGARCH model for the Chinese market. Second, the *β*_1_ parameters of the RealRECH model we used to measure the complex volatility dynamics of time series are statistically significant for all four indices. This means that the RealRECH model can effectively capture the complex volatility dynamics of time series that the RealGARCH model cannot capture, such as long-term dependence and nonlinearity. Finally, the beta and gamma parameters of the RealRECH model are statistically significant for all four indices, demonstrating that the RealRECH model provides the same in-sample economic explainability as the RealGARCH model; that is, it can also explain the clustering of volatility.

In addition, we find that the RealRECH model displays better out-of-sample prediction performance than the RealGARCH model for the CSI 500 and CSI 1000 indices in the Chinese market. The conclusion is the same whether the score indicators we used are calculated based on the demeaned return *y*_*t*_ or on the proxy ${\tilde{\sigma }}_{t}^{2}$ of the real conditional variance *σ*_*t*_^2^. For the SSE 50 and CSI 300 indices in the Chinese market, the RealRECH model displays worse predictive ability than the RealGARCH model. First, past studies have shown that compare to the GARCH model, deep learning yields a significant improvement in predicting the volatility of high-volatility indices [12,13], however, due to the low volatility of the constituent stocks that make up the SSE 50 and CSI 300, the volatility of these two indices is low. Second, the complex dynamics of these two indices may be too weak to capture. Last, in our data analyses, the volatility dynamics evolve in a rapidly forgotten way.

Overall, for the Chinese market, the RealRECH model can better explain in-sample volatility and capture the complex volatility dynamics of time series volatility that the RealGARCH model cannot capture, such as long-term dependence and nonlinearity. In addition, the RealRECH model can better predict the out-of-sample volatility of the CSI 500 and CSI 1000 indices. Zhao et al. [10] found that the SRN-GARCH and SRU-GARCH models can capture the in-sample long-term dependence and nonlinearity of volatility in Chinese stock indices, and they can also be used to predict the out-of-sample volatility of the CSI 500 and CSI 1000 indices better than can the GARCH model. The conclusions in this paper are similar.

Therefore, the innovations of this paper are as follows. First, for the first time, we study the dynamic characteristics of the volatility of different stock indices in the Chinese market using the RealRECH model. The in-sample estimated results suggest that this model not only provides better in-sample volatility explainability than does the RealGARCH model but can also capture the complex dynamics of time series volatility that the RealGARCH model cannot capture, such as long-term dependence and nonlinearity. On the basis of in-sample analysis, we validate the quality of out-of-sample volatility predictions for the Chinese market obtained with the proposed model and find that the predictive ability of the model is superior to that of the RealGARCH model for some indices. Finally, we enrich the research on the RECH model and Chinese market volatility prediction.

The remainder of this paper is organized as follows. Section 2 provides a literature review. Section 3 gives a review of the RealGARCH model, the LSTM model, the measurement of realized volatility, the RealRECH model and its Bayesian inference, and the construction of variables is explained. In Section 4, the data processing steps and descriptive statistics are described. Section 5 presents the empirical analysis, and the conclusions are given in Section 6.

## 2. Literature review

### 2.1 GARCH and its variants

Many studies have used GARCH models for volatility fitting and forecasting. Prajna et al. [14] compared GARCH and GARCH-fractional cointegration based on the price return volatility of several energy commodities and found that GARCH is more suitable for long-memory stationary data and that the GARCH-fractional cointegration model is more suitable for long-memory nonstationary data. Juri and Bogdan [15] proposed a new model, the GARCH in-mean Glosten-Jagannathan-Runkle leverage (GARCH-M-GJR-LEV) model, to capture the asymmetry in variance and return equations. They applied this model to determine whether there is an asymmetric relationship between the risk premium and volatility changes in the S&P 500 market index, and the model they proposed outperformed the GARCH model and its variants. Usman et al. [16] examined the role of oil shocks in predicting U.S. stock market volatility by using the GARCH mixed data sampling (GARCH-MIDAS) model and found that symmetric net price change (SNP) information is most useful for forecasting the volatility of the S&P 500 market index. Gregory et al. [17] forecasted crude oil intraday volatility by using the Functional GARCH (FGARCH) model and the Functional GARCH-X (FGARCH-X) model and found that although the FGARCH-X model performs worse than the FGARCH(1,1) in terms of out-of-sample forecasting, it can capture the long-range dependence and potential seasonality of the West Texas Intermediate (WTI) crude oil commodity, which the FGARCH(1,1) model cannot capture.

There have been many studies of RealGARCH-type models. Wu et al. [18] combined the Realized Exponential GARCH (RealEGARCH) model with skewness and kurtosis and introduced the RealEGARCH-SK model for the VaR forecasting of Chinese stock indices; notably, they found that this model can account for the time-varying skewness and kurtosis of Chinese stock indices. Wang et al. [19] compared the prediction accuracy of the GARCH model, the EGARCH model and the RealGARCH model based on Chinese indices and found that the RealGARCH model yields the best prediction accuracy. Kordbacheh et al. [20] compared the accuracy of VaR forecasting with the GARCH model and the RealGARCH model based on 3 different distributions, and the results of VaR forecasting for the Tehran Stock Exchange index showed that the RealGARCH model is superior.

Dong and Yang [21] found that if volatility derivatives are combined with stock returns in the GARCH model for option pricing, the model yields a highly persistent volatility component, so the leverage effect remains prominent over long horizons. Marcos et al. [22] introduced a class of multivariate GARCH (MGARCH) models for multiasset option pricing and performed a full calibration with three bivariate series of index returns and their corresponding volatility indices in joint maximum likelihood estimation. They found that one of these models fit margin distributions better and improved the overall likelihood estimates.

### 2.2 Neural network models

Neural network models also play an important role in volatility forecasting. Aryan et al. [23] proposed a new recurrent ensemble deep random vector functional link (RedRVFL) network model for financial time series forecasting and compared its forecasting accuracy and predictive capability with those of the LSTM network model, the GRU model, the variational mode decomposition LSTM (VMD-LSTM) model and 9 other models. They found that the RedRVFL network model yields the best forecasting accuracy and predictive capability of all the models based on 11 indices. Nguyen et al. [24] combined the LSTM model and the stochastic volatility (SV) model and proposed the LSTM-SV model to capture the dynamics of the financial volatility process. They found that the model can capture the nonlinear dependence in the latent volatility process and yields better forecasting performance than the SV model. The SV model can also be combined with other models to improve forecasts of the S&P 500 market index and the ASX 200 index. Nguyen et al. [25] proposed the statistical recurrent stochastic volatility (SR-SV) model by combining the SV model with the RNN model to capture the dynamics of stochastic volatility. They found that the SR-SV model can capture nonlinearity and long-memory autodependence, and the model displayed impressive out-of-sample forecasting performance for 5 stock indices. Nybo [12] applied the artificial neural network (ANN) model and the GARCH model for the volatility prediction of the American stock market and compared the performance of these models. The results obtained with three GARCH specifications and three ANN architectures showed that the ANN model is more suitable for stocks with low volatility and that the GARCH model is more suitable for stocks with medium or high volatility. Shaik and Sejpal [13] compared the predictive performance of the ANN model and the GARCH model for the volatility prediction of the Indian stock market and found that the ANN model outperformed the GARCH model in low-volatility periods of the indices, and vice versa.

Lu and Xu [26] proposed a time-series recurrent neural network (TRNN) model for stock price prediction and compared its efficiency and accuracy with those of an RNN model and an LSTM model. They found that the TRNN model achieved better Dow Jones index predictions. Carlos et al. [27] compared the forecasting performance of a simple RNN model, a multilayer perceptron model and an LSTM model based on the S&P 500, DAX, AEX and SMI indices and found that if the fractal and self-similarity behaviors of the indices are considered, the model predictions of the S&P 500 improve. Zhang et al. [28] established the variational modal decomposition sample entropy gated recurrent unit (VMD-SE-GRU) framework to forecast the price of crude oil and found that the new framework produces highly accurate forecasts with a short runtime.

### 2.3 Recurrent conditional heteroscedasticity model and its variants

As mentioned above, although they provide better predictive performance than the GARCH model in many cases, deep learning models lack economic explainability. In response to this shortcoming, Nguyen et al. [29] developed a recurrent conditional heteroscedasticity (RECH) model based on the FNN-GARCH model proposed by Donaldson and Kamstra [30]. Unlike Roh [31], who used the conditional variance of the GARCH model as the input of a neural network, Nguyen et al. designed the RECH model with a simple recurrent neural network added to the GARCH model to enhance volatility predictions on the basis of retaining the ability to explain volatility to economic phenomena. The RECH model has another advantage; that is, it can capture the complex dynamics of volatility that the GARCH model cannot capture, such as long-term dependence and nonlinearity. They found that the RECH model provided better predictions than GARCH-type models for 4 stock indices.

Liu et al. [11] developed the long short-term memory realized generalized autoregressive conditional heteroskedasticity (LSTM-RealGARCH) model, or the RealRECH model, by adding the LSTM structure of Hochreiter and Schmidhuber [32] to the RealGARCH model. They found that the model achieved better predictions of the volatility of multiple stock indices than did the RECH model and RealGARCH model. Specifically, the RealRECH model exhibited better in-sample volatility explainability and out-of-sample predictive ability than did the RECH model and RealGARCH model for 31 non-Chinese stock indices.

## 3. Models

### 3.1 Realized generalized autoregressive conditional heteroskedasticity model

The traditional GARCH model relies on the square of the daily rate of return, which only contains a weak signal of daily volatility *σ*_*t*_^2^. Generally, low-frequency data cannot meet the modeling accuracy demand. Therefore, it is increasingly common for scholars to use high-frequency data to more accurately estimate daily volatility [33–35].

Engle [36] was the first to introduce the realized volatility metric of Andersen and Bollerslev [33] into the GARCH model. Since then, an increasing number of scholars, such as Forsberg and Bollerslev [37], Engle and Gallo [38], Corsi [39] and Shephard and Sheppard [40], have conducted similar studies. The realized GARCH (RealGARCH) model proposed by Hansen et al. [3] is as follows:

$$y_{t}=\sigma_{t}\varepsilon_{t},\;t=1,2\ldots ,T$$ (1)

$${\sigma_{t}}^{2}=\omega +\gamma r\nu_{t-1}+\beta {\sigma_{t-1}}^{2}$$ (2)

$$r\nu_{t}=\xi +\varphi {\sigma_{t}}^{2}+\tau (\varepsilon_{t})+\mu_{t}$$ (3)

where *ε*_*t*_ is independent and identically distributed and obeys the standard normal distribution. *μ*_*t*_ is also independent and identically distributed and obeys a normal distribution with a mean of 0 and a variance of *σ*_*μ*_^2^. *rν*_*t*_ is used to measure the realized volatility, and the function *τ*(*ε*) reflects the different ranges of changes in volatility when there are shocks in different directions, where $\tau (\varepsilon )=\tau_{1}\varepsilon +\tau_{2}(\varepsilon^{2}‐1)$.

### 3.2 Long short-term memory unit

Because the basic RNN model is not flexible enough for modeling in many cases and is difficult to train, many scholars have proposed improved RNN models, such as the LSTM model proposed by Hochreiter and Schmidhuber [32]. This model adopts a gate-like structure to control the retention of data, and the specific form is as follows:

$${g^{i}}_{t}=\sigma (w^{i}[h_{t-1},x_{t}]+b^{i})$$ (4)

$${g^{f}}_{t}=\sigma (w^{f}[h_{t-1},x_{t}]+b^{f})$$ (5)

$${g^{o}}_{t}=\sigma (w^{o}[h_{t-1},x_{t}]+b^{o})$$ (6)

$${\tilde{c}}_{t}=\tanh(w^{c}[h_{t-1},x_{t}]+b^{c})$$ (7)

$$c_{t}={g^{i}}_{t}*{\tilde{c}}_{t}+{g^{f}}_{t}*c_{t-1}$$ (8)

$$h_{t}={g^{o}}_{t}*\tanh(c_{t})$$ (9)

$${\hat{y}}_{t}=g_{y}(w^{y}h_{t}+b^{y})$$ (10)

In a basic RNN, the information stored in the hidden state is fully considering during each iteration. However, this is not the case with LSTM, which can decide how to deal with new information through the memory unit ${\tilde{c}}_{t}$ in (8), and it can retain, forget or update information. This information unit ${\tilde{c}}_{t}$ achieves updating by partially forgetting the information before *c*_*t*−1_ and adding new information from ${\tilde{c}}_{t}$. The degrees to which historical information is forgotten and new information is added are controlled by the forget gate *g*^*f*^_*t*_ and the input gate *g*^*i*^_*t*_, respectively. Finally, the degree of current memory usage of the final output is controlled by the output gate *g*^*o*^_*t*_ [41].

### 3.3 Realized recurrent conditional heteroscedasticity model and realized volatility measurement

In this section, the realized recurrent conditional heteroscedasticity (RealRECH) model, which was proposed by Liu et al. [11], is introduced. Compared with the RealGARCH model, the RealRECH model includes an LSTM structure. Therefore, its modeling flexibility is improved. Additionally, compared with the RECH model, the RealRECH model considers realized volatility and achieves better prediction accuracy. RealRECH(p, q) can be expressed as:

$$y_{t}=\sigma_{t}\varepsilon_{t},\;t=1,2\ldots ,T$$ (11)

$${\sigma_{t}}^{2}=g(\omega_{t})+\sum_{i=1}^{p}\gamma_{i}r\nu_{t-i}+\sum_{j=1}^{q}\beta_{j}{\sigma^{2}}_{t-j}$$ (12)

$$r\nu_{t}=\xi +\varphi {\sigma_{t}}^{2}+\tau (\varepsilon_{t})+\mu_{t}$$ (13)

$$\omega_{t}=LSTM(x_{t})$$ (14)

$$x_{t}=(\omega_{t-1,}y_{t-1},{\sigma_{t-1}}^{2},r\nu_{t-1})$$ (15)

Like RealGARCH, RealRECH also includes the calculation in Eq (13) to reflect the change in realized volatility. The RealRECH model is actually the RealGARCH model with LSTM.

Through the forget and input gates, the RNN *g*(*ω*_*t*_) can quickly adapt to changes in volatility. In periods of high volatility, when the historical volatility is quite different from the current volatility, the volatility changes greatly, and the forget gate is activated, causing the RNN to ignore irrelevant historical information and allowing the RNN to quickly acquire new patterns through the input gate.

In periods when the volatility does not change much, the forget gate is closed, resulting in persistent volatility. In this paper, we only use one realized volatility measure *rν*_*t*_ in the RealRECH model. However, it is easy to incorporate as many realized volatility measures as desired by using them as additional inputs of *X*_*t*_. In this paper, based on Nguyen et al. [27] and Liu et al. [11], only RealRECH(1,1) is considered in the analysis, and the model is expressed as

$$y_{t}=\sigma_{t}\varepsilon_{t},\;t=1,2\ldots ,T$$ (16)

$${\sigma_{t}}^{2}=g(\omega_{t})+\gamma r\nu_{t-1}+\beta {\sigma_{t-1}}^{2}$$ (17)

$$r\nu_{t}=\xi +\varphi {\sigma_{t}}^{2}+\tau (\varepsilon_{t})+\mu_{t}$$ (18)

$$\omega_{t}=\beta_{0}+\beta_{1}h_{t}$$ (19)

$$h_{t}=LSTM(x_{t})$$ (20)

$$x_{t}=(\omega_{t-1},y_{t-1},{\sigma^{2}}_{t-1},r\nu_{t-1})$$ (21)

### 3.4 Bayesian inference of RealRECH

In Bayesian form, the posterior distribution *π*(*θ*) = *p*(*θ*|*y*_1:*T*_) can be expressed as

$$\pi (\theta )=\frac{p(y_{1:T}|\theta )p(\theta )}{p(y_{1:T})}$$ (22)

where *p*(*y*_1:*T*_|*θ*) is the likelihood function, *p*(*θ*) is the prior distribution, and $p(y_{1:T})=\int_{\Theta }p(y_{1:T}|\theta )p(\theta )d\theta$ is the marginal likelihood function. *θ* is composed of recurrent parameters and GARCH parameters.

#### 3.4.1 Sequential Monte Carlo (SMC) method for in-sample analysis

Cui et al. [6] showed that the sequential Monte Carlo (SMC) method is an effective method for Bayesian inference and the prediction of volatility. This method can effectively sample from the nonstandard posterior distribution to conveniently obtain one-step-ahead predictions and marginal likelihood values.

In sampling from a posterior distribution *π*(*θ*) in the SMC method [41–43], M weighted particles ${\left\{W_{0}^{j},\theta_{0}^{j}\right\}}_{j=1}^{M}$ are first extracted from an easy-to-sample distribution *π*_0_(*θ*), and the particles are then traversed using the intermediate distribution *π*_t_(*θ*),*t* = 1,…,*K*, which becomes the posterior distribution *π*(*θ*), i.e., *π*_K_(*θ*) = *π*(*θ*). To simplify the calculation, *π*_0_(*θ*) is set to the prior distribution *p*(*θ*), that is, *π*_0_(*θ*) = *p*(*θ*).

For the construction of intermediate distribution sequences, Neal [42] proposed likelihood annealing, and Chopin [43] proposed data annealing. In general, likelihood annealing is suitable for in-sample analysis, and the corresponding SMC sequence is constructed as follows:

$$\pi_{t}(\theta )≔\pi_{t}(\theta |y_{1:T})\propto p{\left(y_{1:T}|\theta \right)}^{\gamma_{t}}p(\theta ),$$ (23)

where *γ*_*t*_ satisfies $0=\gamma_{0}<\gamma_{1}<\ldots <\gamma_{K}=1$.

The SMC algorithm usually consists of three steps: reweighting, resampling, and Markov movement. Specifically, in the t-th cycle, the weighted particles ${\left\{W_{t-1}^{j},\theta_{t-1}^{j}\right\}}_{j=1}^{M}$ used to approximate the intermediate distribution *π*_t-1_(*θ*) are reweighted to approximate the target distribution *π*_t_(*θ*). For particle resampling, the effective sample size (ESS) proposed by Kass et al. [44] is used to evaluate the validity of *π*_t_(*θ*) weighted particles. When the ESS falls below a certain value, the particles are resampled. These equally weighted sample particles are then updated with a Markov kernel based on an invariant distribution *π*_t_(*θ*). The SMC algorithm with likelihood annealing is described in Appendix A.1

#### 3.4.2 Sequential Monte Carlo (SMC) method for out-of-sample analysis and parameter setting

For out-of-sample rolling predictions, the SMC algorithm with data annealing developed by Chopin [43] is most suitable for updating the parameter values of the model according to the new information available. The weighted particles produced by the SMC sampling algorithm obey the following distribution sequence:

$$\pi_{t}(\theta )≔\pi_{t}(\theta |y_{1:t})\propto p(y_{1:t}|\theta )p(\theta )\propto \pi_{t-1}(\theta )p(y_{t}|\theta ,y_{1:t-1})$$ (24)

where *y*_1:*t*_ denotes the data available at t. The unnormalized weights at step t of the SMC process in the SMC algorithm with likelihood annealing change to the following form:

$$w_{t}^{i}=W_{t-1}^{j}\frac{p{\left(y_{1:T}|\theta_{t-1}^{j}\right)}^{\gamma_{t}}p(\theta_{t-1}^{j})}{p{\left(y_{1:T}|\theta_{t-1}^{j}\right)}^{\gamma_{t-1}}p(\theta_{t-1}^{j})}=W_{t-1}^{j}p{\left(y_{1:T}|\theta_{t-1}^{j}\right)}^{\gamma_{t}-\gamma_{t-1}},\;j=1,\ldots ,M$$ (25)

Appendix A.2 describes the SMC algorithm with data annealing.

In this paper, an SMC algorithm with likelihood annealing is used for in-sample Bayesian analysis, and an SMC algorithm with data annealing is used for out-of-sample prediction. Table 1 lists the parameter values used in the SMC sampling algorithm. The parameter settings are based on those of Nguyen et al. [25]:

**Table 1:** Parameter settings of the SMC algorithm.

Variable	Description	Value
K	The number of likelihood annealing iterations	10000
M	The number of particles	2000
c	A multiple of the ESS threshold value	0.8
**N**_**lik**_	Number of Markov movement steps used by the SMC algorithm with likelihood annealing	20
**N**_**data**_	Number of Markov movement steps used by the SMC algorithm with data annealing	15

### 3.5 Construction of out-of-sample predictive ability indicators

In this paper, we construct four out-of-sample prediction ability indicators based on the demeaned return *y*_*t*_: the partial prediction score (PPS), the number of violations (#Vio), the quantile score (QS), and the hit rate (%Hit). For the test data *D*_*test*_, the number of observed samples is *T*_*test*_, and the estimated mean value of the posterior parameters *θ* is $\hat{\theta }$. In this paper, the PPS calculation method proposed by Gneiting and Raftery [45] is adopted as follows:

$$PPS=-\frac{1}{T_{test}}\sum_{D_{test}}\log p(y_{t}|y_{1:t-1},\hat{\theta })$$ (26)

The lower the PPS is, the better the prediction of the model. #Vio is defined as the number of violations for test dataset *D*_*test*_, where the observed value *y*_*t*_ is outside the 99% interval of the one-step prediction.

A major application of volatility models is predicting the VaR. Taylor [46] used the QS to measure the performance of a model for predicting the VaR. The QS is defined as follows:

$$QS=\frac{1}{T_{test}}\sum_{D_{test}}\left(\alpha -I_{y_{t}\leq q_{t,\alpha }})(y_{t}-q_{t,\alpha }\right)$$ (27)

where *q*_*t*,*α*_ is the conditional *α*-VAR-predicted value of *y*_*t*_ based on *y*_1:*t*−1_, and *α*-VAR is defined as the *α* quantile of the one-step-ahead prediction value based on distribution $p(y_{t}|y_{1:t-1},\hat{\theta })$. The smaller the value of QS is, the better the predictive ability of the model for the VaR. Taylor [46] defined %Hit as the proportion of test data for which *y*_*t*_ is lower than the predicted value of *α*-VAR. When the model predictions are accurate, the %Hit value is close to *α*.

The above four scoring metrics are complementary. For example, when adjusting the model so that #Vio decreases, the PPS and QS usually increase. Overall, the closer to *α* the %Hit value of the volatility prediction model is and the smaller the other three scoring metrics are, the better the out-of-sample prediction ability of the model.

In addition, we establish six other scoring metrics based on the proxy ${\tilde{\sigma }}_{t}^{2}$ for real conditional variance *σ*_*t*_^2^, namely, two mean square errors (MSEs), two mean absolute errors (MAEs), quasilikelihood (QLIKE) and R2LOG, to test the out-of-sample prediction performance of the RealRECH model; notably, the prediction performance is assessed by comparing the predicted volatility with the ex post realized volatility. We use part of the test dataset *D*_*test*_, denoted as *T*_*test*_, in these calculations, which are expressed as follows:

$$MSE_{1}=T_{test}^{-1}\sum_{D_{test}}{\left(\sigma_{t}-{\hat{\sigma }}_{t}\right)}^{2}$$ (28)

$$MSE_{2}=T_{test}^{-1}\sum_{D_{test}}{\left(\sigma_{t}^{2}-{\hat{\sigma }}_{t}^{2}\right)}^{2}$$ (29)

$$MAE_{1}=T_{test}^{-1}\sum_{D_{test}}\left|\sigma_{t}-{\hat{\sigma }}_{t}\right|$$ (30)

$$MAE_{2}=T_{test}^{-1}\sum_{D_{test}}\left|\sigma_{t}^{2}-{\hat{\sigma }}_{t}^{2}\right|$$ (31)

$$QLIKE=T_{test}^{-1}\sum_{D_{test}}\left(\log({\hat{\sigma }}_{t}^{2})+\sigma_{t}^{2}{\hat{\sigma }}_{t}^{-2}\right)$$ (32)

$$R^{2}LOG=T_{test}^{-1}\sum_{D_{test}}{\left[\log(\sigma_{t}^{2}{\hat{\sigma }}_{t}^{-2})\right]}^{2}$$ (33)

where ${\hat{\sigma }}_{t}$ is the one-step rolling window prediction of potential *σ*_*t*_ and ${\tilde{\sigma }}_{t}$ is the square root of the adjusted true variance. The ex post volatility index used in this paper is the realized variance (rv), and Eqs (28)–(33) reflect the out-of-sample prediction ability of the model based on the proxy ${\tilde{\sigma }}_{t}^{2}$ of real conditional variance *σ*_*t*_^2^. The smaller the indicator values are, the better the out-of-sample prediction ability of the model.

## 4. Data processing and descriptive statistics

To study the in-sample interpretation and out-of-sample prediction abilities of the RealGARCH and RealRECH models based on the SSE 50, CSI 300, CSI 500 and CSI 1000 indices in the Chinese market, we use the daily closing price {*P*_*t*_,*t* = 1,…,*T*_*P*_} to calculate the daily average rate of return:

$$y_{t}=100(\log\frac{P_{t+1}}{P_{t}}-\frac{1}{T_{P}-1}\sum_{i=1}^{T_{P}-1}\log\frac{P_{i+1}}{P_{i}}),\;t=1,2,\ldots ,T_{P}-1$$ (34)

In this paper, we use the method proposed by Andersen and Bollerslev [33] to calculate the realized variance (rv) and the proxy ${\tilde{\sigma }}_{t}^{2}$ of the true conditional variance *σ*_*t*_^2^. In this calculation, the daily realized variance is calculated by using the daily 5-minute data. We use {*P*_*t*,*i*_,*i* = 1,…,*T*_*t*_} to express the 5-minute closing price on the Tth-day, and the formulas for the realized variance and the real conditional variance on the Tth-day are as follows:

$$rv_{t}=\sum_{i=1}^{T_{t}-1}{\left(\log\;P_{t,j+1}-\log\;P_{t,j}\right)}^{2}+{\left(\log\;P_{t,1}-\log\;P_{t-1,T_{t}}\right)}^{2},\;t=1,2,\ldots ,T_{P}-1$$ (35)

$${\tilde{\sigma }}_{t}^{2}=\hat{c}\cdot rv_{t},\;where\;\hat{c}=\frac{T_{\mathrm{out}}^{‐1}\sum_{t=T_{in}+1}^{T}{\left[y_{t}-E(y_{t}|I_{t-1})\right]}^{2}}{T_{\mathrm{out}}^{‐1}\sum_{t=T_{in}+1}^{T}rv_{t}},\;t=T_{in}+1,2,\ldots ,T$$ (36)

where $P_{t-1,T_{t}}$ is the closing price of the previous day.

In the selection of data samples, we use a total of 1500 demeaned returns and realized variances of these four indices from March 20, 2015, to May 17, 2021, as in-sample data and use a total of 500 demeaned returns and realized variances from May 18, 2021, to June 6, 2023, as out-of-sample data. This means that for in-sample analysis, day 0 is March 20, 2015, and day T_in_ is May 17, 2021. For out-of-sample analysis, at time t, the window we used for parameter setting to forecast volatility at time t+1 is from day 0 to day T_in_+t, and dynamic forecasting is performed with a recursive window.

Table 2 reports the descriptive statistics of the average returns of the four indices and the results of the modified range based on a standard deviation (R/S) test [47]. Modified R/S models, such as those of Lo [47], Giraitis et al. [48] and Breidt et al. [49], are often used to test whether the square of financial time series returns has long-term memory. V(q) is the test value of the lag Q period of the modified R/S of Lo. In the last three columns of Table 2, for all the four indices, the values in the upper and lower rows are the test values of the absolute demeaned rate of return and the square of the demeaned rate of return used in Lo’s modified R/S test, respectively; additionally, an asterisk indicates that the difference is statistically significant at the 5% level.

**Table 2:** Descriptive statistical information and R/S test of Lo’s correction.

Variables	Minimum	Maximum	Standard deviation	Skewness	Kurtosis	V (10)	V (20)	V (30)
SSE 50	−9.850	7.549	1.417	−0.598	9.268	2.576*	2.074*	1.811*
						2.529*	2.095*	1.843*
CSI 300	−9.154	6.499	1.429	−0.872	9.043	2.672*	2.124*	1.846*
CSI 300	−9.154	6.499	1.429	−0.872	9.043	2.650*	2.165*	1.893*
CSI 500	−9.075	6.399	1.660	−1.116	8.371	3.133*	2.455*	2.112*
CSI 500	−9.075	6.399	1.660	−1.116	8.371	2.974*	2.382*	2.075*
CSI 1000	−9.190	6.422	1.788	−0.997	7.135	3.171*	2.486*	2.136*
CSI 1000	−9.190	6.422	1.788	−0.997	7.135	3.038*	2.436*	2.113*

Table 2 shows that all the demeaned return series of the indices exhibit a certain negative skewness, high kurtosis and various levels of volatility. Compared with the other three sets of demeaned returns, the demeaned returns of the CSI 500 are more skewed. The kurtosis of SSE 50 is greater than that of the other three indices, and the volatility of CSI 1000 is greater than that of the other three indices. The results of three Lo’s modified R/S tests for assessing the long-term dependence in the lag Q period show that there is long-term dependence in the demeaned returns of the SSE 50, CSI 300, CSI 500 and CSI 1000, and it is statistically significant at the 5% level. Therefore, for in-sample analysis, it is appropriate to use the RealGARCH and RealRECH models to fit the in-sample volatility of these indices, and it is also appropriate to test their out-of-sample volatility prediction ability.

## 5. Empirical analysis results

### 5.1 In-sample estimation results of RealGARCH and RealRECH

Table 3 gives the in-sample estimation results of the RealGARCH and RealRECH models for the demeaned returns of the four indices. The mean and standard deviation of the posterior estimation are obtained by using the SMC algorithm with likelihood annealing. First, when the volatility of these four indices is fitted with the RealRECH model, if measured by the marginal likelihood value (llh) of each index, the levels of volatility explainability of the RealGARCH and RealRECH models are different. The marginal likelihood values of the RealRECH model for the four indices are all greater than those of the RealGARCH model, indicating that the in-sample volatility explainability of the RealRECH model is greater than that of the RealGARCH model for all four indices.

**Table 3:** In-sample estimation results of the RealGARCH and RealRECH models.

Variables	Beta	Gamma	Beta0	Beta1	Xi	Phi	Tau1	Tau2	Sigma2u	Llh
SSE50										
RealGARCH	**0.648**	**0.364**	**0.111**		**0.109**	**0.809**	**−0.397**	**0.538**	**10.308**	**−**2462.1
	(0.022)	(0.031)	(0.023)		(0.075)	(0.051)	(0.081)	(0.044)	(0.354)	(0.042)
RealRECH	**0.627**	**0.284**	**0.037**	**0.818**	**0.037**	**0.973**	**−0.41**	**0.606**	**9.612**	**−**2452*
	(0.025)	(0.024)	(0.024)	(0.167)	(0.034)	(0.046)	(0.076)	(0.041)	(0.331)	(1.367)
CSI300										
RealGARCH	**0.712**	**0.299**	**0.082**		**0.125**	**0.807**	**−0.518**	**0.601**	**11.208**	**−**2468.9
	(0.022)	(0.028)	(0.02)		(0.082)	(0.051)	(0.083)	(0.045)	(0.386)	(0.041)
RealRECH	**0.708**	**0.199**	**0.026**	**0.948**	**0.026**	**1.031**	**−0.52**	**0.735**	**9.677**	**−**2455.4*
	(0.021)	(0.017)	(0.013)	(0.132)	(0.021)	(0.044)	(0.076)	(0.047)	(0.307)	(0.795)
CSI500										
RealGARCH	**0.689**	**0.315**	**0.202**		**0.067**	**0.803**	**−0.561**	**0.631**	**15.327**	**−**2724.7
	(0.027)	(0.032)	(0.03)		(0.058)	(0.045)	(0.104)	(0.053)	(0.506)	(0.031)
RealRECH	**0.691**	**0.262**	**0.077**	**0.875**	**0.051**	**0.869**	**−0.614**	**0.685**	**14.324**	**−**2720.6*
	(0.029)	(0.028)	(0.048)	(0.166)	(0.05)	(0.042)	(0.099)	(0.05)	(0.502)	(0.919)
CSI1000										
RealGARCH	**0.71**	**0.272**	**0.278**		**0.056**	**0.815**	**−0.658**	**0.672**	**19.92**	**−**2841
	(0.03)	(0.032)	(0.041)		(0.053)	(0.044)	(0.117)	(0.063)	(0.655)	(0.024)
RealRECH	**0.68**	**0.258**	**0.088**	**0.944**	**0.052**	**0.85**	**−0.685**	**0.718**	**19.242**	**−**2832*
	(0.033)	(0.031)	(0.056)	(0.193)	(0.05)	(0.045)	(0.116)	(0.066)	(0.599)	(3.235)

Note: The estimated means and standard deviations of the parameters are provided in the table. The standard deviations are provided in brackets. The estimated mean value of each parameter is given bold when it exceeds the statistical level of 5% of the estimated standard deviation.

* indicates that Llh is larger than that of the RealGARCH model. The last column shows the estimated log-marginal likelihood with the Monte Carlo standard deviation in parentheses, averaged over 10 different runs of the SMC with likelihood annealing.

Second, in the RealRECH model, the *β*_1_ parameter used to measure the dynamics of the complex volatility of time series is significant for all four indices. Thus, among the four selected indices, the RealRECH model can effectively capture the complex dynamics of time series volatility, which the RealGARCH model cannot capture, such as long-term dependence and nonlinearity. Finally, the beta and gamma parameters of RealRECH are significant for all four indices, revealing that the RealRECH model provides the same level of economic explainability as the RealGARCH model; that is, it can also explain the clustering of volatility.

In addition, from other parameters such as xi, phi, tau1, tau2 and sigma2u, it is clear that the RealRECH model retains the volatility explainability of the RealGARCH model.

**Fig 1:**
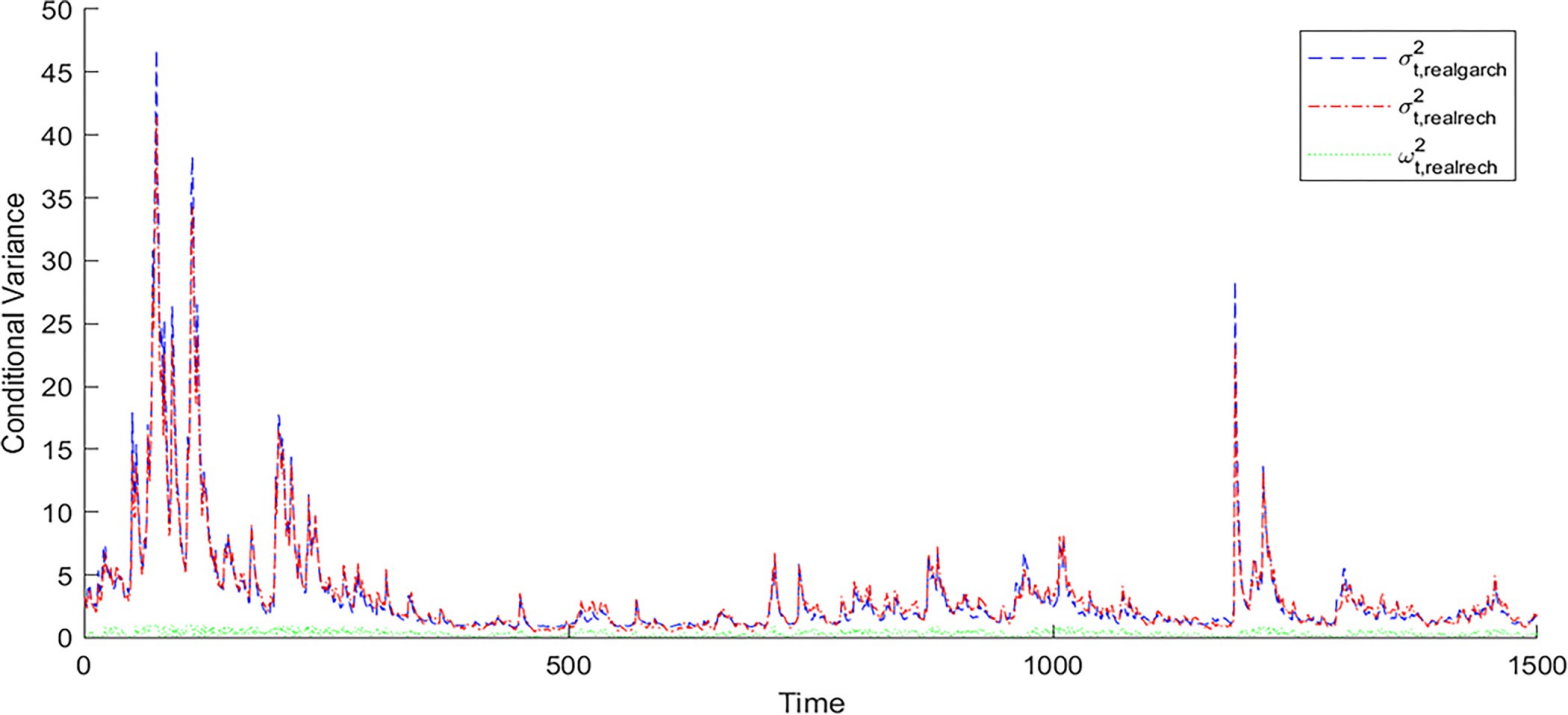
Conditional variance of the RealGARCH model and the conditional variance and the recurrent component *ω*_*t*_ of the RealRECH model for the CSI 500.

Fig 1 depicts the in-sample conditional variance of the RealGARCH model, the in-sample conditional variance of the RealRECH model and the in-sample recurrent part *ω*_*t*_ of the CSI 500 index, and Fig B.1.1 in S1 Appendix shows the same for the CSI 1000. As shown in Fig 1 and Fig B.1.1 in S1 Appendix, for the CSI 500 and CSI 1000 indices, the recurrent part *ω*_*t*_ of the RealRECH model captures the dynamic changes in the non-RealGARCH part of the volatility; that is, the predictions are low in the low-volatility period and high in the high-volatility period. In other words, *ω*_*t*_ is large in periods of high volatility for the two indices, and the RealRECH model captures the complex dynamics of this volatility well.

Figs B.1.2 and B.1.3 in S1 Appendix are similar in-sample diagrams for the SSE 50 and CSI 300 indices, respectively. Fig B.1.2 in S1 Appendix shows that for the SSE 50 index, the recurrent part *ω*_*t*_ of the RealRECH model does not respond to the dynamic change in volatility and is similar to a constant. First, the complex dynamics of the volatility of this index may be weak. Second, based on our data, the volatility dynamics evolve in a rapidly forgotten way, and historical information is quickly forgotten. Fig B.1.3 in S1 Appendix shows that for the CSI 300 index, the recurrent part of the *ω*_*t*_ RealRECH model only responds to dynamic changes in volatility to some extent; the reasons are the same as those for the SSE 50.

**Fig 2:**
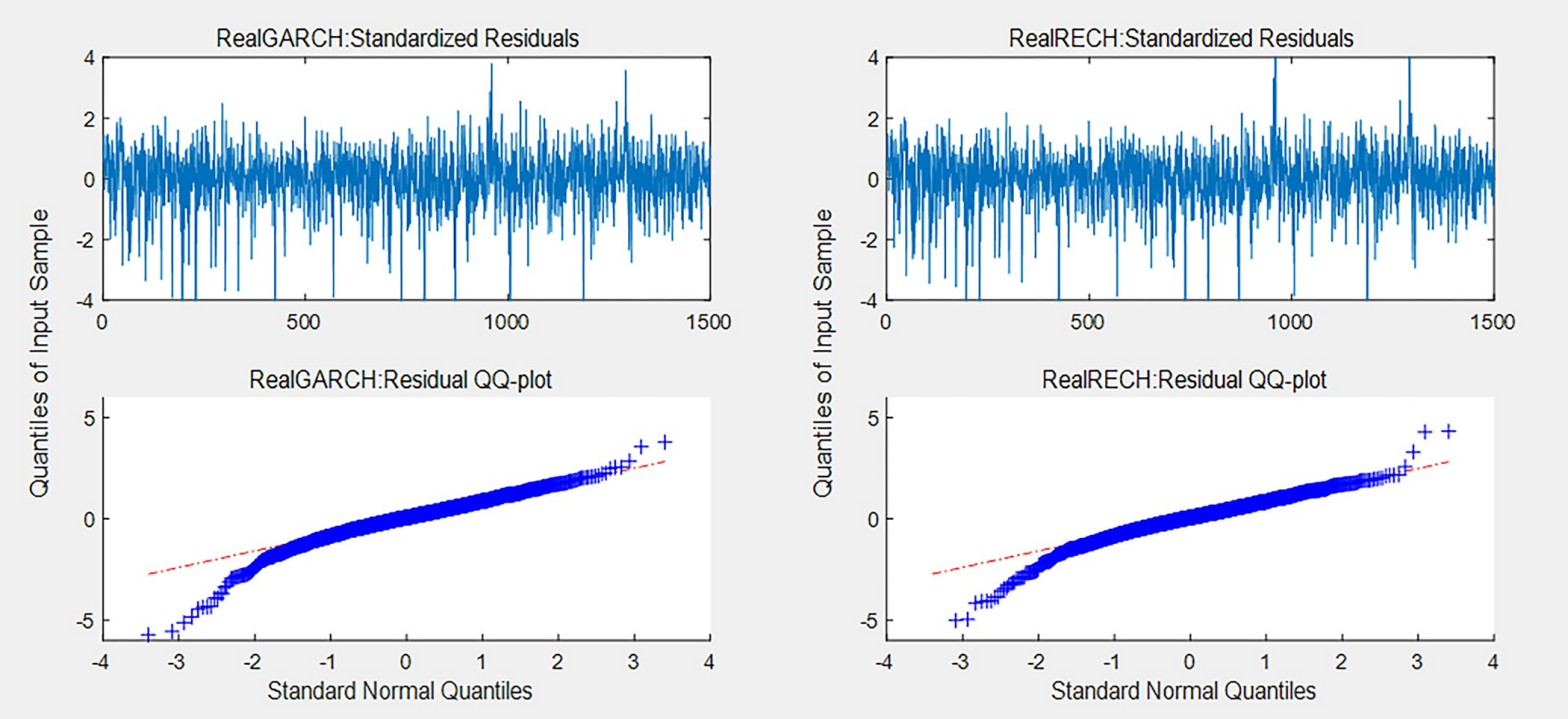
Estimation residuals ${\hat{\varepsilon }}_{t}$ and QQ plots of the RealGARCH and RealRECH models for the CSI 500.

Fig 2 shows the estimated residuals ${\hat{\varepsilon }}_{t}$ and quantile–quantile (QQ) plots of the RealGARCH and RealRECH models for the demeaned returns in the CSI 500. Figs B.2.1-B.2.3 in S1 Appendix show the same results for the CSI 1000, SSE 50 and CSI 300, respectively. As shown in Fig 2 and Figs B.2.1-B.2.3 in S1 Appendix, in general, the estimated residuals of the RealRECH model are closer to the standard normal distribution than those of the RealGARCH model, and the residual points in the QQ plots are close to the 45° line.

The in-sample analysis shows that the RealRECH model outperforms the RealGARCH model when fitting the volatility using the in-sample data from the four indices and displays the same volatility explainability when fitting the in-sample data.

### 5.2 Out-of-sample estimation results of RealGARCH and RealRECH

Pagan and Schwert [50] and Donaldson and Kamstra [30] showed that better in-sample performance does not result in better out-of-sample performance. Therefore, to compare whether the out-of-sample prediction ability of the RealRECH model is better than that of the RealGARCH model based on the four indices in the Chinese market, we obtain values of the four out-of-sample indicators based on the demeaned returns *y*_*t*_ in Table 4. Table 5 presents the values of six out-of-sample prediction ability indicators based on the proxy ${\tilde{\sigma }}_{t}^{2}$ of the real conditional variance *σ*_*t*_^2^.

**Table 4:** Out-of-sample prediction performance measured based on yt.

	PPS	#Vio	QS	Hit	Count
SSE 50					
RealGARCH	1.5442*	5*	0.0382*	0.0099*	
	(0)	(0)	(0)	(0)	
RealRECH	1.5514	7.1	0.0402	0.0163	(0, 4)
	(0.0008)	(0.3162)	(0.0002)	(0.0008)	
CSI 300					
RealGARCH	1.4791*	7	0.0356	0.016*	
	(0)	(0)	(0)	(0)	
RealRECH	1.4941	6.1*	0.0356	0.0182	(1, 2)
	(0.0027)	(0.3162)	(0.0006)	(0.0031)	
CSI 500					
RealGARCH	1.4861	7	0.0415	0.0179	
	(0)	(0)	(0)	(0)	
RealRECH	1.4661*	6.5*	0.0391*	0.016*	(4, 0)
	(0.0028)	(0.527)	(0.0003)	(0)	
CSI 1000					
RealGARCH	1.6637	4.5*	0.0467	0.016	
	(0)	(0.527)	(0)	(0)	
RealRECH	1.654*	6.1	0.0456*	0.014*	(3, 1)
	(0.0052)	(0.7378)	(0.0006)	(0.0016)	

Note: The table provides the estimated mean and standard deviation of the measures from 10 runs, and the standard deviation is in parentheses. The left (right) Count value reflects the number of model score metrics that are better than (worse than) those of the RealGARCH model.

* indicates that the score is better than that of the RealGARCH model.

**Table 5:** Out-of-sample prediction performance measured based on the proxy of the real conditional variance.

	MSE_1_	MSE_2_	MAE_1_	MAE_2_	QLIKE	R^2^LOG	Count
SSE 50							
RealGARCH	0.1548*	1.5857*	0.2968*	0.7544*	1.8796	0.4109*	
	(0)	(0.0004)	(0)	(0.0002)	(0.0008)	(0.0001)	
RealRECH	0.1567	1.6222	0.304	0.7723	1.8584*	0.416	(1, 5)
	(0.0014)	(0.0094)	(0.0027)	(0.0065)	(0.0448)	(0.0068)	
CSI 300							
RealGARCH	0.1342*	1.1879*	0.2798*	0.6731*	1.7896*	0.4114*	
	(0)	(0.0004)	(0)	(0.0002)	(0.0008)	(0.0002)	
RealRECH	0.1525	1.2908	0.3109	0.7478	1.965	0.4791	(0, 6)
	(0.004)	(0.0381)	(0.0061)	(0.0151)	(0.061)	(0.0151)	
CSI 500							
RealGARCH	0.1875	1.7755	0.3545	0.8582	2.3645	0.6365	
	(0)	(0.0005)	(0.0001)	(0.0002)	(0.001)	(0.0003)	
RealRECH	0.1807*	1.6896*	0.3438*	0.8404*	2.2436*	0.5944*	(6, 0)
	(0.0036)	(0.0264)	(0.004)	(0.0112)	(0.0327)	(0.0104)	
CSI 1000							
RealGARCH	0.2468	3.7264	0.3966	1.1323	2.559	0.596	
	(0.0001)	(0.0012)	(0.0001)	(0.0004)	(0.0011)	(0.0003)	
RealRECH	0.226*	3.4834*	0.3736*	1.0868*	2.4185*	0.5196*	(6, 0)
	(0.006)	(0.0382)	(0.0082)	(0.0227)	(0.0523)	(0.0205)	

Note: The table provides the estimated mean and standard deviation of the measures from 10 runs, and the standard deviation is in parentheses. The left (right) Count value reflects the number of model score metrics that are better than (worse than) those of the RealGARCH model.

* indicates that the score is better than that of the RealGARCH model.

Table 4 shows the prediction scores of PPS, #Vio, QS and %Hit for the two models based on the demeaned returns *y*_*t*_. As shown in Table 4, for the out-of-sample performance of the CSI 500 and CSI 1000, the prediction ability of the RealRECH model is better than that of the RealGARCH model; notably, the three prediction scores, namely, the PPS, #Vio and QS, are lower for the RealRECH model than for the RealGARCH model, and the %Hit of the model is close to 0.01, the *α* value we set in the paper. Thus, the model yields good prediction ability for these two indices. In terms of the out-of-sample performance of the SSE 50 and CSI 300 models, for the same standard, the out-of-sample prediction ability of the RealRECH model is worse than that of the RealGARCH model.

As we mentioned above, past studies have shown that compare to the GARCH model, deep learning yields a significant improvement in predicting the volatility of high-volatility indices [12,13], however, due to the low volatility of the constituent stocks that make up the SSE 50 and CSI 300, the volatility of these two indices is low. In addition, the recurrent parts *ω*_*t*_ of the SSE 50 and CSI 300 indices show that their complex volatility dynamics may be too weak to capture and their volatility dynamics evolve in a rapidly forgotten way. These factors could reflect why the RealRECH model displays poor out-of-sample prediction ability for the SSE 50 and CSI 300 indices and good prediction ability for the CSI 500 and CSI 1000 indices.

Our conclusion is similar to that of Zhao et al. [10], who noted that the SRN-GARCH and SRU-GARCH models displayed better prediction ability than the GARCH model for only the CSI 500 and CSI 1000 indices.

Table 5 presents the values of the six out-of-sample volatility prediction performance indicators defined in Eqs (28)–(33) for the two models, measured based on the proxy ${\tilde{\sigma }}_{t}^{2}$ of the real conditional variance *σ*_*t*_^2^. As shown in Table 5, compared with RealGARCH model, the RealRECH model displays better out-of-sample prediction ability for the CSI 500 and CSI 1000 indices. Specifically, for the CSI 1000 index, the RealRECH model yields the greatest improvement in out-of-sample prediction ability because of the large gaps in the indicators between the two models, and for the CSI 500 index, the RealRECH model produces a certain improvement in out-of-sample prediction ability. However, for the SSE 50 and CSI 300 indices, although the RealRECH model exhibits better volatility explainability than the RealGARCH model, it does not achieve better prediction ability in the out-of-sample analysis.

Tables 3–5 show that the RealRECH model better explains in-sample volatility based on the four indices and can capture the complex dynamics of time series volatility that the RealGARCH model cannot, such as long-term dependence and nonlinearity. According to the out-of-sample performance, the RealRECH model has a better ability to predict volatility only for the CSI 500 and CSI 1000.

**Fig 3:**
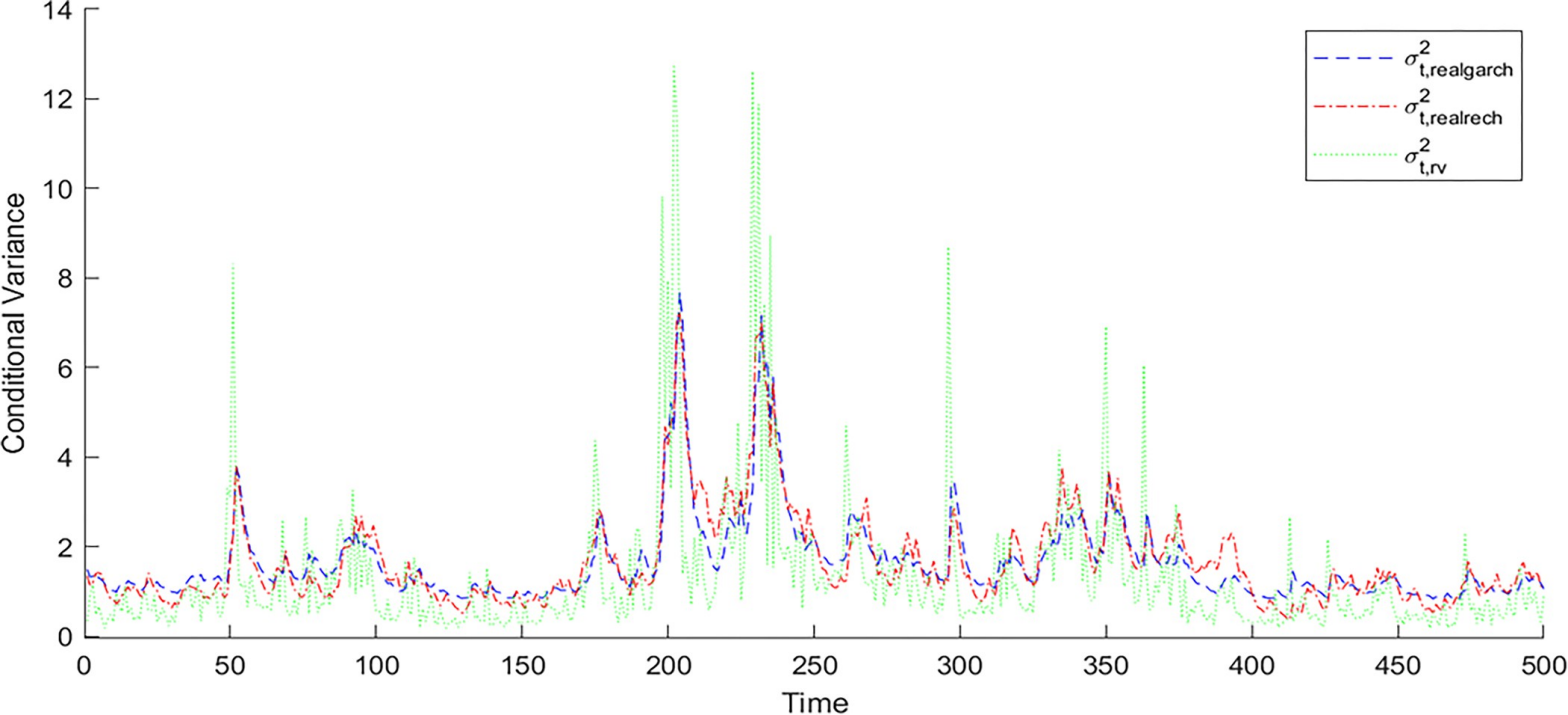
One-step-ahead prediction of the conditional variance of the RealGARCH and RealRECH models and the adjusted value of the realized variance for the CSI 500.

Fig 3 shows a comparison of the predicted values of the conditional variance and the adjusted value of the realized variance in the corresponding period between the RealGARCH and RealRECH models for the CSI 500 stock index. Figs B.3.1-B.3.3 in S1 Appendix show the same for the CSI 1000, SSE 50 and CSI 300, respectively. First, in general, the RealGARCH model can track the realized variance in the SSE 50 and CSI 300 well, and the RealRECH model can track the realized variance in the CSI 500 and CSI 1000 well.

Moreover, for two stock indices, CSI 500 and CSI 1000, in terms of tracking their realized variance, the overall prediction ability of the RealRECH model is better than that of the RealGARCH model, and the corresponding predictions are closer to the adjusted values of the realized variance than are those of the RealGARCH model.

For the other two stock indices, the SSE 50 and CSI 300, the RealRECH model only shows better prediction ability than the RealGARCH model in a few periods, and the prediction of volatility is generally worse than that of the RealGARCH model. The reasons are stated above.

## 6. Conclusion

Traditional volatility and deep learning models are mainly used to study the volatility of Chinese stock indices in the literature. There have been few studies of the ability of the RECH model to predict the volatility of the Chinese market, and there have been no studies on the use of high-frequency data to fit and predict the volatility of the Chinese market using the RealRECH model. In this paper, we use the RealRECH model for both fitting and prediction for the first time, and the results are compared with those of the RealGARCH model to determine whether the RealRECH model can improve predictions of Chinese stock index volatility.

In this paper, we divide the four stock indices in China, the SSE 50, CSI 300, CSI 500 and CSI 1000, into in-sample data and out-of-sample data and analyze them to evaluate the performance of the RealRECH model in the Chinese market. The in-sample empirical results show that first, the RealRECH model has a greater marginal likelihood than does the RealGARCH model in terms of four selected indices, reflecting stronger volatility explainability. Second, in the RealRECH model, the *β*_1_ parameter used to measure the complex dynamics of time series is statistically significant according to the four indices; notably, this parameter allows the model to capture the complex dynamics of time series volatility that the RealGARCH model cannot capture, such as long-term dependence and nonlinearity. Finally, the beta and gamma parameters of the RealRECH model are statistically significant among the four indices, indicating that the RealRECH model has the same economic explainability as the RealGARCH model; that is, it can also explain the clustering of volatility. The recurrent parts *ω*_*t*_ of the SSE 50 and CSI 300 are either nearly constant or minimally variable. First, the complex dynamics of these two indices may be too weak to capture. Second, in our data, their volatility dynamics evolve in a rapidly forgotten way.

The results of the out-of-sample predictions show that for the CSI 500 and CSI 1000 indices, the RealRECH model achieves better prediction ability, and the adjusted value of the realized variance is closer to the real variances than that of the RealGARCH model. For the SSE 50 and CSI 300, the prediction ability of the RealRECH model is worse than that of the RealGARCH model. The reasons are the same. In addition, past studies have shown that deep learning yields a more significant improvement in predicting the dynamics of high-volatility indices compared to the GARCH model, however, due to the low volatility of the constituent stocks that make up the SSE 50 and CSI 300, the volatility of these two indices is low.

In short, for the Chinese market, the RealRECH model displays better in-sample volatility explainability and can capture the complex dynamics of time series volatility that the RealGARCH model cannot capture; additionally, the out-of-sample prediction ability of the RealRECH model is better for the CSI 500 and CSI 1000 than that of the RealGARCH model. Therefore, for the Chinese market, it is highly suitable to use the RealRECH model for fitting and predicting the CSI 500 and CSI 1000.

## Supporting information

S1 Appendix (DOC)

S1 Data Data1. (ZIP)

S2 Data Data2&code. (ZIP)

## References

[1] Engle R.F., Autoregressive conditional heteroscedasticity with estimates of the variance of United Kingdom inflation, Econometrica 50 (1982) 987–1007. 10.2307/1912773.

[2] Bollerslev T., Generalized autoregressive conditional heteroskedasticity, J. Econom. 31 (1986) 307–327. 10.1016/0304-4076(86)90063-1.

[3] Hansen P.R., Huang Z., Shek H.H., Realized GARCH: A joint model for returns and realized measures of volatility, J. Appl. Econom. 27 (2012) 877–906. 10.1002/jae.1234.

[4] Liu Y., Novel volatility forecasting using deep learning–long short term memory recurrent neural networks, Expert Syst. Appl. 132 (2019) 99–109. 10.1016/j.eswa.2019.04.038.

[5] Bucci A., Realized volatility forecasting with neural networks, J. Financ. Econom. 18 (2020) 502–531. 10.1093/jjfinec/nbaa008.

[6] Cui Y., Li L., Tang Z., Risk analysis of China stock market during economic downturns–based on GARCH-VaR and wavelet transformation approaches, Asian Econ. Financ. Rev. 11 (2021) 322–336. 10.18488/journal.aefr.2021.114.322.336.

[7] Liu M., Lee C.-C., Choo W.-C., The role of high-frequency data in volatility forecasting: Evidence from the China stock market, Appl. Econ. 53 (2021) 2500–2526. 10.1080/00036846.2020.1862747.

[8] Liu T., Shi Y., Innovation of the component GARCH model: Simulation evidence and application on the Chinese stock market, Mathematics 10 (2022) 1903. 10.3390/math10111903.

[9] Luo Y. Comparing recurrent neural network with GARCH model on forecasting volatility based on SSE 50ETF. InSecond International Conference on Statistics, Applied Mathematics, and Computing Science (CSAMCS 2022) 2023 Mar 28 (Vol. 12597, pp. 729–735). SPIE. 10.1117/12.2673039.

[10] Zhao H., Zhang G., Yan X., Fan R., Predicting the volatility of Chinese stock indices based on recurrent conditional heteroskedasticity. Working paper, (2024). 10.1371/journal.pone.0308967 PMC11488709 39423217

[11] Liu C., Wang C., Tran M., Kohn R., Realized recurrent conditional heteroskedasticity model for volatility modelling, (2023). https://doi.org/arXiv:2302.08002.

[12] Nybo C. (2021). Sector Volatility Prediction Performance Using GARCH Models and Artificial Neural Networks. https://arxiv.org/abs/2110.08499.

[13] Shaik M., & Sejpal A. (2020). The Comparison of GARCH and ANN Model for Forecasting Volatility: Evidence based on Indian Stock Markets: Predicting Volatility using GARCH and ANN Models. The Journal of Prediction Markets, 14(2), 103–121. 10.5750/jpm.v14i2.1843.

[14] Prajna Pramita Izati Dedy Dwi Prastyo, Muhammad Sjahid Akbar, Modeling the Volatility of World Energy Commodity Prices Using the GARCH-Fractional Cointegration Model, Procedia Computer Science, Volume 234, 2024, Pages 412–419, ISSN 1877-0509, 10.1016/j.procs.2024.03.022.

[15] Trifonov Juri, Potanin Bogdan, GARCH-M model with an asymmetric risk premium: Distinguishing between ‘good’ and ‘bad’ volatility periods, International Review of Financial Analysis, Volume 91, 2024, 102941, ISSN 1057-5219, 10.1016/j.irfa.2023.102941.

[16] Ghani Usman, Zhu Bo, Ghani Maria, Khan Nasir, Raja Danish Akbar khan, Role of oil shocks in US stock market volatility: A new insight from GARCH-MIDAS perspective, Resources Policy, Volume 85, Part B, 2023, 103933, ISSN 0301-4207, 10.1016/j.resourpol.2023.103933.

[17] Rice Gregory, Wirjanto Tony, Zhao Yuqian, Exploring volatility of crude oil intraday return curves: A functional GARCH-X model, Journal of Commodity Markets, Volume 32, 2023, 100361, ISSN 2405-8513, 10.1016/j.jcomm.2023.100361.

[18] Wu Xinyu, Xia Michelle, Zhang Huanming, Forecasting VaR using realized EGARCH model with skewness and kurtosis, Finance Research Letters, Volume 32, 2020, 101090, ISSN 1544-6123, 10.1016/j.frl.2019.01.002.

[19] Susheng W. A. N. G., Guanglu L. I., & Junbo W. A. N. G. (2023). Volatility Prediction Evaluation of GARCH Models Based on Loss Functions. Operations Research and Management Science, 32(9), 101. http://www.jorms.net/CN/10.12005/orms.2023.0291

[20] Kordbacheh H., Zabol M. A., & Abounoori E. (2023). Forecasting Daily Value-at-Risk of the Tehran Stock Exchange Index using Realized GARCH Approach. Journal of Economic Research and Policies, 31(105), 65–88. http://qjerp.ir/article-1-2667-en.html.

[21] Dong Hwan Oh Yang-Ho Park, GARCH option pricing with volatility derivatives, Journal of Banking & Finance, Volume 146, 2023, 106718, ISSN 0378-4266, 10.1016/j.jbankfin.2022.106718.

[22] Marcos Escobar-Anel Javad Rastegari, Stentoft Lars, Covariance dependent kernels, a Q-affine GARCH for multi-asset option pricing, International Review of Financial Analysis, Volume 87, 2023, 102622, ISSN 1057-5219, 10.1016/j.irfa.2023.102622.

[23] Bhambu Aryan, Gao Ruobin, Ponnuthurai Nagaratnam Suganthan, Recurrent ensemble random vector functional link neural network for financial time series forecasting, Applied Soft Computing, Volume 161, 2024, 111759, ISSN 1568-4946, 10.1016/j.asoc.2024.111759.

[24] Nguyen N., Tran M. N., Gunawan D., & Kohn R. (2019). A long short-term memory stochastic volatility model. https://arxiv.org/abs/1906.02762.

[25] Nguyen T. N., Tran M. N., Gunawan D., & Kohn R. (2023). A statistical recurrent stochastic volatility model for stock markets. Journal of Business & Economic Statistics, 41(2), 414–428. 10.1080/07350015.2022.2028631.

[26] Lu Minrong, Xu Xuerong, TRNN: An efficient time-series recurrent neural network for stock price prediction, Information Sciences, Volume 657, 2024, 119951, ISSN 0020-0255, 10.1016/j.ins.2023.119951.

[27] Mendoza Carlos, Kristjanpoller Werner, Minutolo Marcel C., Market index price prediction using Deep Neural Networks with a Self-Similarity approach, Applied Soft Computing, Volume 146, 2023, 110700, ISSN 1568-4946, 10.1016/j.asoc.2023.110700.

[28] Zhang Shiqi, Luo Jing, Wang Shuyuan, Liu Feng, Oil price forecasting: A hybrid GRU neural network based on decomposition–reconstruction methods, Expert Systems with Applications, Volume 218, 2023, 119617, ISSN 0957-4174, 10.1016/j.eswa.2023.119617.

[29] Nguyen T.-N., Tran M.-N., Kohn R., Recurrent conditional heteroskedasticity, J. Appl. Econom. 37 (2022) 1031–1054. 10.1002/jae.2902.

[30] Donaldson R.G., Kamstra M., An artificial neural network-GARCH model for international stock return volatility, J. Empir. Finance 4 (1997) 17–46. 10.1016/S0927-5398(96)00011-4.

[31] Roh T.H., Forecasting the volatility of stock price index, Expert Syst. Appl. 33 (2007) 916–922. 10.1016/j.eswa.2006.08.001.

[32] Hochreiter S., Schmidhuber J., Long short-term memory, Neural Comput. 9 (1997) 1735–1780. doi: 10.1162/neco.1997.9.8.1735 9377276

[33] Andersen T.G., Bollerslev T., Answering the skeptics: Yes, standard volatility models do provide accurate forecasts, Int. Econ. Rev. 39 (1998) 885–905. 10.2307/2527343.

[34] Barndorff-Nielsen O., Power and bipower variation with stochastic volatility and jumps, J. Financ. Econom. 2 (2004) 1–37. 10.1093/jjfinec/nbh001.

[35] Barndorff-Nielsen O.E., Hansen P.R., Lunde A., Shephard N., Designing realized kernels to measure the ex post variation of equity prices in the presence of noise, Econometrica 76 (2008) 1481–1536. 10.3982/ECTA6495.

[36] Engle R., New frontiers for arch models, J. Appl. Econom. 17 (2002) 425–446. 10.1002/jae.683.

[37] Forsberg L., Bollerslev T., Bridging the gap between the distribution of realized (ECU) volatility and ARCH modelling (of the Euro): The GARCH-NIG model, J. Appl. Econom. 17 (2002) 535–548. 10.1002/jae.685.

[38] Engle R.F., Gallo G.M., A multiple indicators model for volatility using intra-daily data, J. Econom. 131 (2006) 3–27. 10.1016/j.jeconom.2005.01.018.

[39] Corsi F., A simple approximate long-memory model of realized volatility, J. Financ. Econom. 7 (2009) 174–196. 10.1093/jjfinec/nbp001.

[40] Shephard N., Sheppard K., Realising the future: Forecasting with high-frequency-based volatility (HEAVY) models, J. Appl. Econom. 25 (2010) 197–231. 10.1002/jae.1158.

[41] Goodfellow I., Bengio Y., A. Courville, Deep Learning, MIT press, Cambridge, MA, 2016.

[42] Neal R.M., Annealed importance sampling, Stat. Comput. 11 (2001) 125–139. 10.1023/A:1008923215028.

[43] Chopin N., A sequential particle filter method for static models, Biometrika 89 (2002) 539–551. 10.1093/biomet/89.3.539.

[44] Kass R.E., Carlin B.P., Gelman A., Neal R.M., Markov chain monte carlo in practice: A roundtable discussion, Am. Stat. 52 (1998) 93–100. 10.2307/2685466.

[45] Gneiting T., Raftery A.E., Strictly proper scoring rules, prediction, and estimation, J. Am. Stat. Assoc. 102 (2007) 359–378. 10.1198/016214506000001437.

[46] Taylor J.W., Forecasting value at risk and expected shortfall using a semiparametric approach based on the asymmetric laplace distribution, J. Bus. Econ. Stat. 37 (2019) 121–133. 10.1080/07350015.2017.1281815.

[47] Lo A.W., Long-term memory in stock market prices, Econometrica 59 (1991) 1279–1313. 10.2307/2938368.

[48] Giraitis L., Kokoszka P., Leipus R., Teyssière G., Rescaled variance and related tests for long memory in volatility and levels, J. Econom. 112 (2003) 265–294. 10.1016/S0304-4076(02)00197-5.

[49] Breidt F.J., Crato N., de Lima P., The detection and estimation of long memory in stochastic volatility, J. Econom. 83 (1998) 325–348. 10.1016/S0304-4076(97)00072-9.

[50] Pagan A.R., Schwert G.W., Alternative models for conditional stock volatility, J. Econom. 45 (1990) 267–290. 10.1016/0304-4076(90)90101-X.

