# Supplementary material for: Predicting the volatility of Chinese stock indices based on realized recurrent conditional heteroskedasticity
Source: PLoS One. 2024 Oct 18;19(10):e0308967. doi: 10.1371/journal.pone.0308967 (PMC11488709; doi:10.1371/journal.pone.0308967)
Supplement: S1 Appendix — (DOC) [file pone.0308967.s001.doc]

**Appendix**

*A.1 The SMC algorithm with likelihood annealing*

1. Sample particles $\theta_{0}^{j}\sim p(\theta)$ and set weights $W_{0}^{j}=1/M,\text{ }j=1,...,M$

2. For $t=1,...,K$,

Step 1: Resampling: Calculate the nonregularization weights

$$w_{t}^{i}=W_{t-1}^{j}\frac{p(y_{1:T}|\theta_{t-1}^{j})^{\gamma_{t}}p(\theta_{t-1}^{j})}{p(y_{1:T}|\theta_{t-1}^{j})^{\gamma_{t-1}}p(\theta_{t-1}^{j})}=W_{t-1}^{j}p(y_{1:T}|\theta_{t-1}^{j})^{\gamma_{t}-\gamma_{t-1}},\text{ }j=1,...,M$$

and the regularization weights

$$W_{t}^{j}=\frac{w_{t}^{j}}{\sum_{s=1}^{M} w_{t}^{s}},\text{ }j=1,...,M$$

Step 2: Calculate the effective sample size (ESS):

$$ESS=\frac{1}{\sum_{j=1}^{M} (W_{t}^{j})^{2}}$$

If $ESS<cM,0<c<1$, then

2.1 Resampling: Sample from particles $\{\theta_{t-1}^{j}\}_{j=1}^{M}$ with weights $\{W_{t}^{j}\}_{j=1}^{M}$, and set $W_{t}^{j}=1/M,\text{ }j=1,...,M$ to obtain new equally weighted particles $\{W_{t}^{j},\theta_{t}^{j}\}_{j=1}^{M}$.

2.2 Markov Movement: For $j=1,...,M$, move particle $\theta_{t}^{j}$ according to the $N_{lik}$ steps of the Metropolis–Hasting random walk method.

2.2 (a) According to the multivariate normal distribution $N(\theta_{t}^{j},\sum_{t})$, generate the proposed particle $\theta_{t}^{j'}$, where is the covariance matrix.

2.2 (b) Let $\theta_{t}^{j}=\theta_{t}^{j'}$ by probability. Then,

$\min\left(1,\frac{p(y_{1:T}|\theta_{t}^{j'})^{\gamma_{t}}p(\theta_{t}^{j'})}{p(y_{1:T}|\theta_{t}^{j})^{\gamma_{t}}p(\theta_{t}^{j})}\right)$,

Otherwise, let $\theta_{t}^{j}$ remain unchanged.

3 Calculate the logarithmic marginal likelihood

$$\log\hat{p}(y_{1:T})=\sum_{t=1}^{K} \log\left(\sum_{j=1}^{M} w_{t}^{j}\right)$$

*A.2 The SMC algorithm with data annealing*

1. Sample particles $\theta_{0}^{j}\sim p(\theta)$ and set weights $W_{0}^{j}=1/M,\text{ }j=1,...,M$

2. For $t=1,...,T$,

Step 1: Resampling: Calculate the nonregularization weights

$$w_{t}^{i}=W_{t-1}^{j}p(y_{t}|y_{1:t-1},\theta_{t-1}^{j}),\text{ }j=1,...,M$$

and the regularization weights

$$W_{t}^{j}=\frac{w_{t}^{j}}{\sum_{s=1}^{M} w_{t}^{s}},\text{ }j=1,...,M$$

Step 2: Calculate the effective sample size (ESS):

$$ESS=\frac{1}{\sum_{j=1}^{M} (W_{t}^{j})^{2}}$$

If $ESS<cM,0<c<1$, then

2.1 Resampling: Sample from weighted particles $\{W_{t}^{j},\theta_{t-1}^{j}\}_{j=1}^{M}$, and set $W_{t}^{j}=1/M,\text{ }j=1,...,M$ to obtain new equally weighted particles $\{W_{t}^{j},\theta_{t}^{j}\}_{j=1}^{M}$.

2.2 Markov Movement: For $j=1,...,M$, move particle $\theta_{t}^{j}$ according to the $N_{data}$ steps of the Metropolis–Hasting random walk method.

2.2 (a) According to the multivariate normal distribution $N(\theta_{t}^{j},\sum_{t})$, generate the proposed particle $\theta_{t}^{j'}$, where is the covariance matrix.

2.2 (b) Let $\theta_{t}^{j}=\theta_{t}^{j'}$ by probability. Then,

$\min\left(1,\frac{p(y_{1:t}|\theta_{t}^{j'})p(\theta_{t}^{j'})}{p(y_{1:t}|\theta_{t}^{j})p(\theta_{t}^{j})}\right)$,

Otherwise, let $\theta_{t}^{j}$ remain unchanged.

*B FIGURES FOR the SSE 50, CSI 300, and CSI 1000*


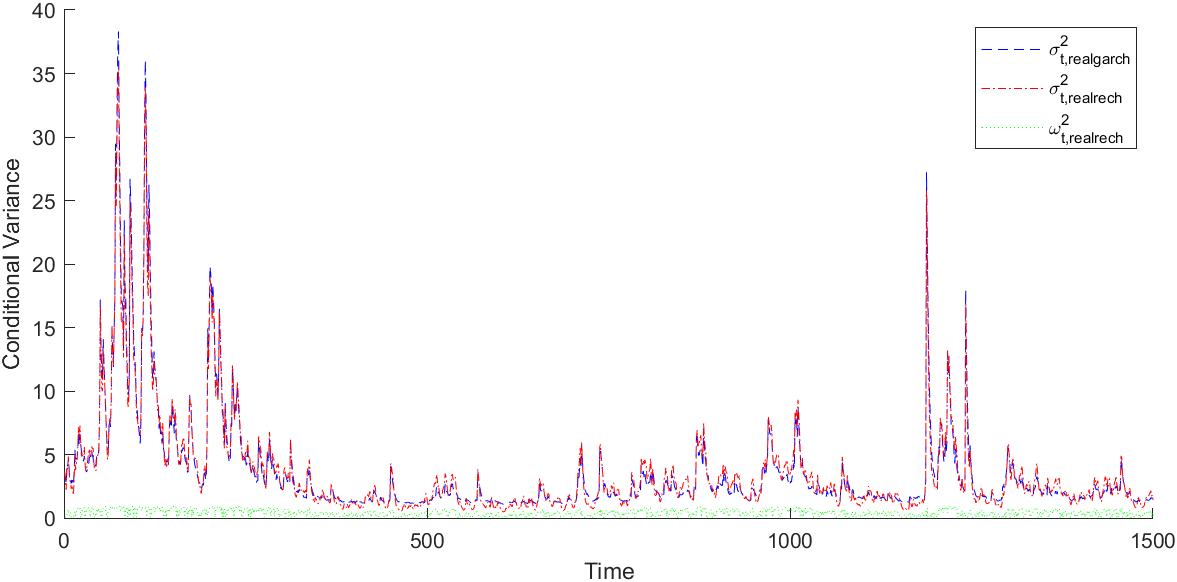


**Fig. B.1.1 Conditional variance of the RealGARCH model and the conditional variance and the recurrent component $\omega_{t}$ of the RealRECH model for the CSI 1000**


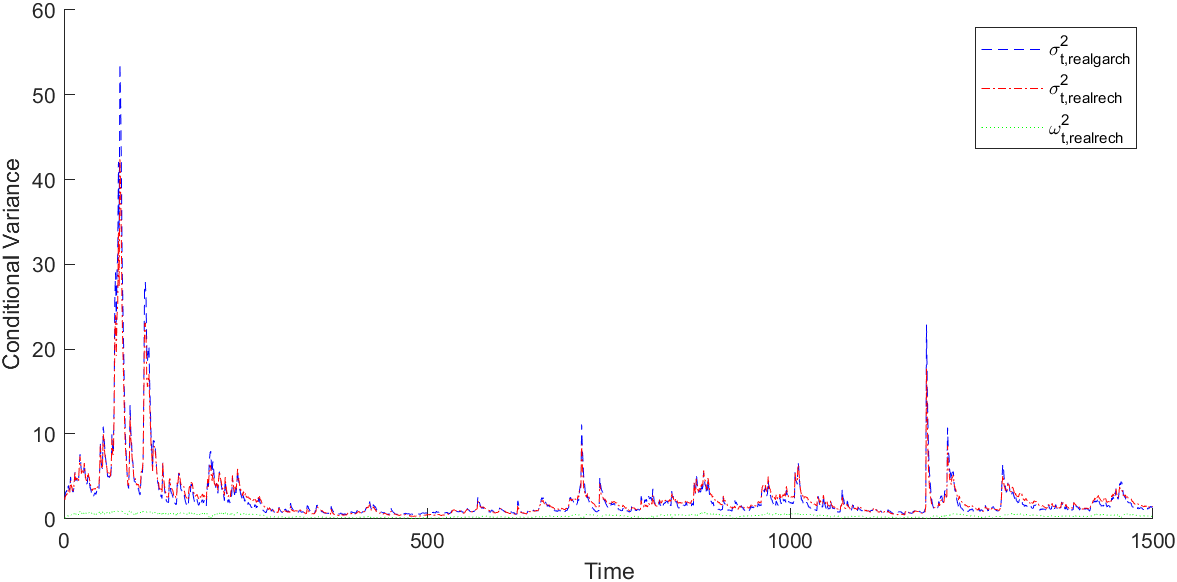


**Fig. B.1.2 Conditional variance of the RealGARCH model and the conditional variance and the recurrent component $\omega_{t}$ of the RealRECH model for the SSE 50**


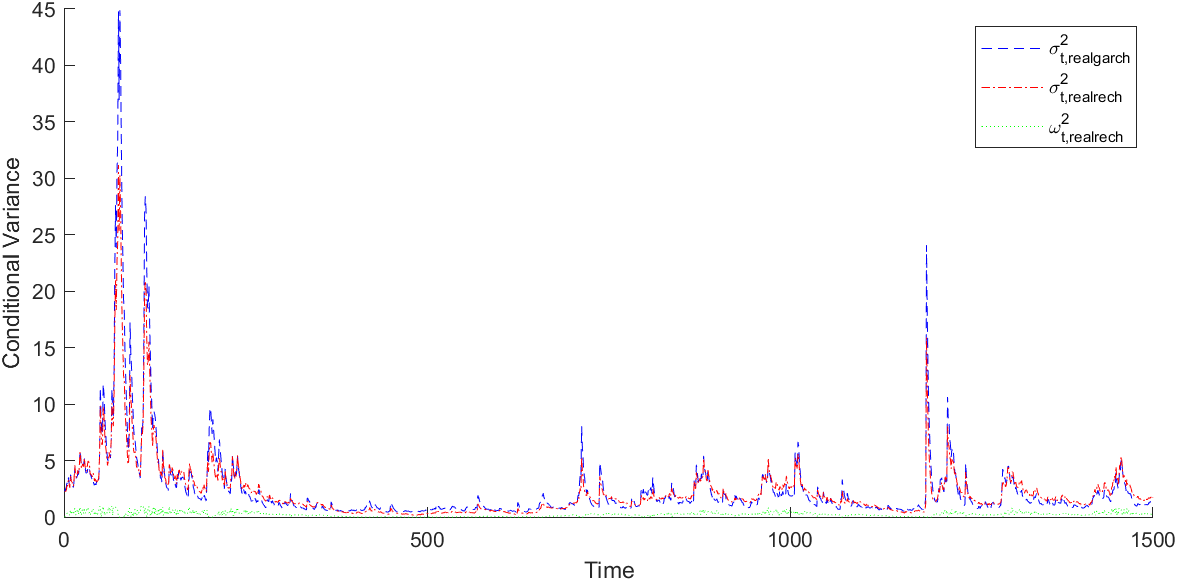


**Fig. B.1.3 Conditional variance of the RealGARCH model and the conditional variance and the recurrent component $\omega_{t}$ of the RealRECH model for the CSI 300**

**Fig. B.2.1 Estimation residuals $\hat{\varepsilon}_{\text{t}}$ and QQ plots of the RealGARCH and RealRECH models for the CSI 1000**

**Fig. B.2.2 Estimation residuals $\hat{\varepsilon}_{\text{t}}$ and QQ plots of the RealGARCH and RealRECH models for the SSE 50**

**Fig. B.2.3 Estimated residuals $\hat{\varepsilon}_{\text{t}}$ and QQ plots of the RealGARCH and RealRECH models for the CSI 300**


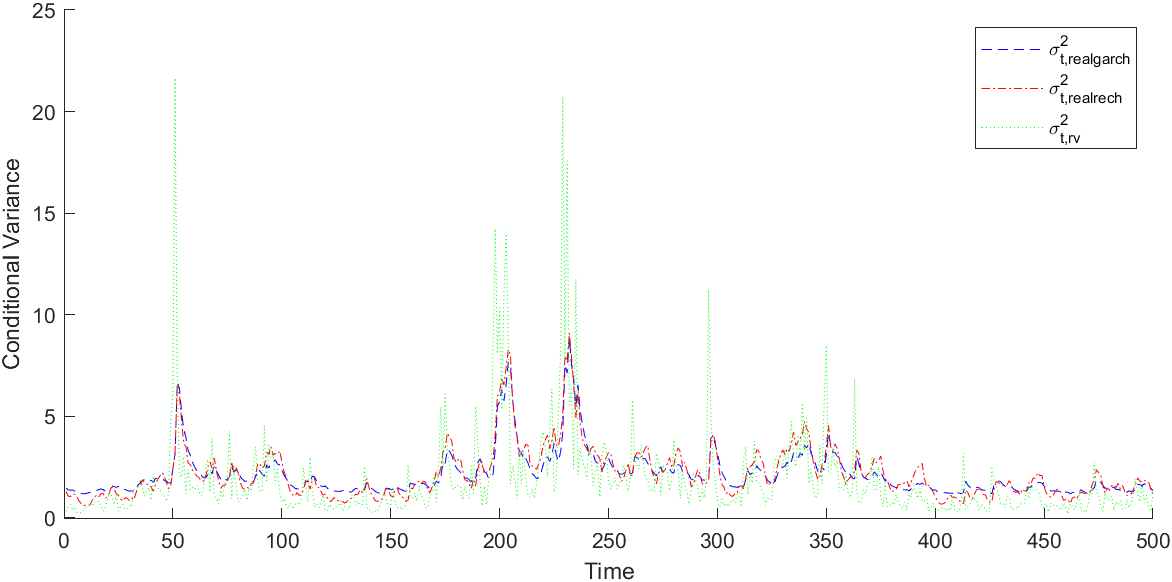


**Fig. B.3.1 One-step-ahead prediction of the conditional variance of the RealGARCH and RealRECH models and the adjusted value of the realized variance for the CSI 1000**


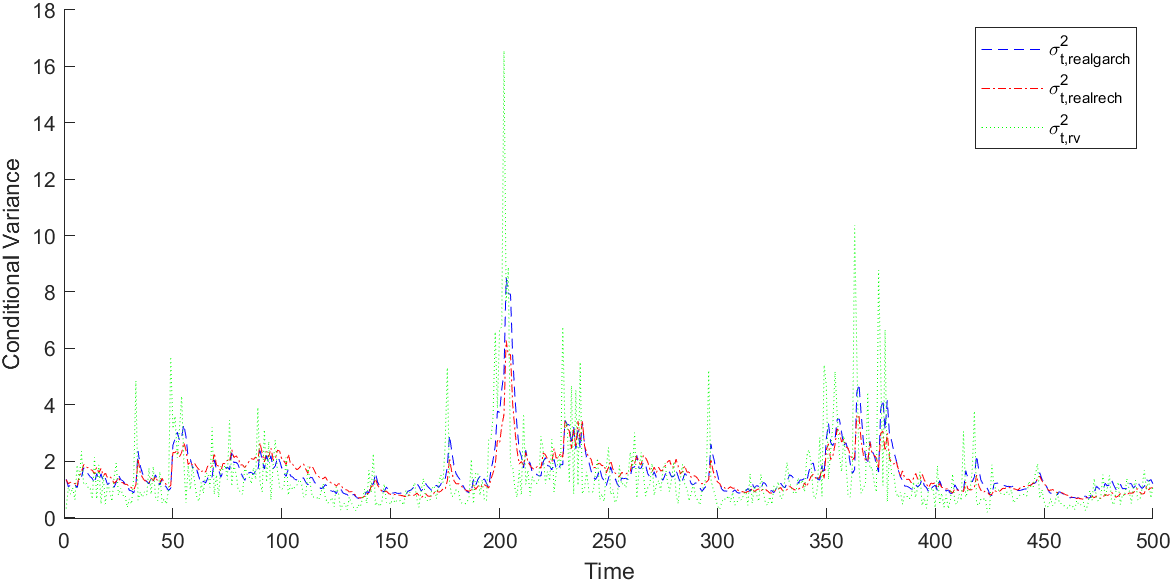


**Fig. B.3.2 One-step-ahead prediction of the conditional variance of the RealGARCH and RealRECH models and the adjusted value of the realized variance for the SSE 50**


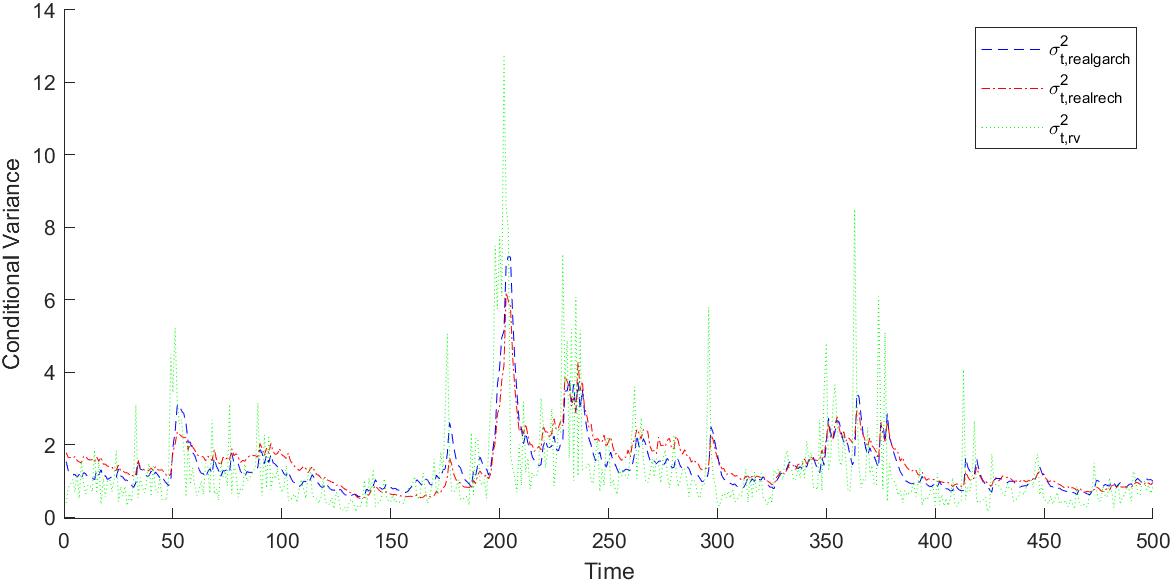


**Fig. B.3.3 One-step-ahead prediction of the conditional variance of the RealGARCH and RealRECH models and the adjusted value of the realized variance for the CSI 300**
